# Comparison of Disability-Adjusted Life Years (DALYs) and Economic Burden on People With Drug-Susceptible Tuberculosis and Multidrug-Resistant Tuberculosis in Korea

**DOI:** 10.3389/fpubh.2022.848370

**Published:** 2022-04-05

**Authors:** SeungCheor Lee, Moon Jung Kim, Seung Heon Lee, Hae-Young Kim, Hee-Sun Kim, In-Hwan Oh

**Affiliations:** ^1^Department of Preventive Medicine, School of Medicine, Kyung Hee University, Seoul, South Korea; ^2^Division of Pulmonary, Sleep and Critical Care Medicine, Department of Internal Medicine, Korea University Ansan Hospital, Ansan-si, South Korea; ^3^Department of Population Health, New York University Grossman School of Medicine, New York, NY, United States; ^4^Department of Health Policy Research, National Evidence-Based Healthcare Collaborating Agency, Seoul, South Korea; ^5^Department of Medicine (AgeTech-Service Convergence Major), School of Medicine, Kyung Hee University, Seoul, South Korea

**Keywords:** tuberculosis, drug-susceptible tuberculosis, multidrug-resistant tuberculosis, disability-adjusted life years, economic disease burden, South Korea

## Abstract

In the future, tuberculosis (TB) will place a heavy burden on the aging population in Korea. To prepare for this crisis, it is important to analyze the disease burden trend of drug-susceptible tuberculosis (DS-TB) and multidrug-resistant tuberculosis (MDR-TB). Measuring disability-adjusted life years (DALYs) and economic burden on MDR-TB patients can help reduce the incidence of TB. Accordingly, in this study, we measured the DALYs and economic burden on DS-TB and MDR-TB patients in 2014–2017 using a combination of National Health Insurance claims data, Annual Report on the Notified TB data, and Statistics Korea's mortality data. The incidence-based DALY approach implemented involved the summation of years of life lost and years lived with disability. For measuring economic burden, direct and indirect costs incurred by patients were totaled. From 2014 to 2017, DALYs per 100,000 people with DS-TB were 56, 49, 46, and 40, respectively, and DALYs per 100,000 people with MDR-TB were 3, 2, 2, and 2, respectively. The economic burden for the DS-TB population from 2014 to 2017 was $143.89 million, $136.36 million, $122.85 million, and $116.62 million, respectively, while that for MDR-TB was $413.44 million, $380.25 million, $376.46 million and $408.14 million, respectively. The results showed a decreasing trend in DALYs and economic burden for DS-TB, whereas MDR-TB was still found to be burdensome without a specific trend. With respect to age, the economic burden for both DS-TB and MDR-TB was higher among men than among women till ≤ 79 years. Conversely, the economic burden for women aged ≥80 years was higher as compared to their male counterparts. In conclusion, the incidence and spread of TB in all areas of society must be suppressed through intensive management of MDR-TB in the older population. We hope that the national TB management project will proceed efficiently when the infectious disease management system is biased to one side due to the COVID-19 pandemic.

## Introduction

Tuberculosis (TB) is a highly infectious disease (1, 2) that has led to the deaths of approximately one billion people worldwide over the past two centuries (3). However, TB is not expected to lead to a global public health crisis by the year 2035, as anticipated by the global strategy for the second half of TB. The situation is improving, although very slowly. The goal of the World Health Organization (WHO) was to reduce the incidence of TB by 4% per year between 2015 and 2020. However, according to a global TB report in 2020, the incidence of the disease decreased by about 2% per year during this period. Similarly, the mortality reduction target set at 7% per year was not achieved, with the achievement of a 3% decrease in mortality from 2015 to 2020 (4).

Meanwhile, drug-resistant TB (DR-TB) has emerged as an important target for TB control worldwide. Drug resistant TB can stem from improper management including poor compliance and wrong treatment as well as from direct transmission. Multidrug-resistant tuberculosis (MDR-TB) refers to tuberculosis that is resistant to at least both isoniazid and rifampicin and extensively drug-resistant tuberculosis (XDR-TB) is an extensively drug-resistant TB that is resistant to any fluoroquinolone and at least one of three injectable second-line drugs in addition to MDR-TB resistance, on drug-susceptibility testing (DST) microbiologically (5). Confronting the threat of COVID-19, we are unsure if the recently decreased drug-susceptible tuberculosis (DS-TB) and MDR-TB prevalence is due to lack of accurate information about real TB patients or improved quarantine policies of social distancing adopted for infectious TB (6).I n 2019, it was reported that about 465,000 TB patients developed resistance to rifampicin, the most effective drug for TB, of which 78% had MDR-TB (7). MDR-TB has been observed in most countries around the world. It is more prominent in 30 countries where the economic burden is the highest on people with MDR-TB (8).

South Korea has high TB incidence and mortality rates. The incidence of TB in South Korea is the highest among the Organization for Economic Cooperation and Development (OECD) countries (9). The plausible reasons for the high burden of TB in South Korea could be as follows. (1) high prevalence of latent TB infection in the elderly after the Korean War (1950–1953), (2) an increasing population with diabetes, (3) previous inadequate TB patient management, (4) immigrants from high-burden countries, and (5) characteristics of M. tuberculosis strain of Beijing lineage (10). Additionally, there is a high risk of recurrence and incidence of MDR-TB all over the world (11). In 2017, a total of 36,044 TB patients were reported in Korea, of which 681 were infected with MDR-TB; in addition, 1,816 TB deaths were reported (12). Furthermore, Korea is experiencing rapid aging. Moreover, TB mortality in the older population (individuals aged over 80) increased by ~35% from 2001 to 2016 (13).

Based on these statistics, TB will put a heavy burden on Korea in the future. To prepare for this crisis, it is important to analyze the disease burden trends of DS-TB and MDR-TB based on the age and sex of the affected population (14–17). The need to strengthen the management of MDR-TB has emerged as an effective approach to the reduction of TB incidence. Since 2005, diagnosis and treatment of active TB had been fully supported by the extended national health insurance benefit coverage, classified as severe disease. TB case monitoring and financial support were reinforced since 2009. In addition, starting with TB close contacts since 2015, latent TB was also included to the extended national health insurance category. Moreover, for the MDR-TB patients, financial support for the living expenses and prior deliberation process for the new MDR-TB drugs have begun (18). Recently, efforts for improving medication adherence with digital health initiatives such as video observation treatment (VOT) are being tried (19), and prior deliberation process for the new MDR-TB drugs enhanced prescription of new TB drugs, funded by government. Still, further public attention is needed to help Korean doctors to be aware of government's recommendation regarding drug usage as well as treatment of TB.

There is a lack of research for the measurement of health outcomes of MDR-TB patients with the use of a standardized methodology as a result of limited data sources and difficulties in selecting subjects. In this study, National Health Insurance (NHI)-integrated data can be used as a source of information for the calculation and comparison of the DALYs of and economic burden on TB patients with different levels of drug resistance to generate reliable evidence. Therefore, this research aimed to identify the current disease burden and trends of DS-TB and MDR-TB in South Korea and calculate the economic burden on patients utilizing NHI-integrated data from 2014 to 2017.

## Materials and Methods

### Data Source and Participants

This study was conducted using a combination of the Annual Report on the Notified TB data, NHI claims data, and Statistics Korea's mortality data. The Annual Report on the Notified TB data was prepared to analyze information pertaining to TB patients diagnosed or treated at public health centers and hospitals across the country and to plan, implement, and evaluate national TB management policies based on the results. The data were collected through the integrated disease and health management system, NHI claims data refers to a sample research data, customized research data, and health disease indicators based on evidence accumulated from the NHIS. Statistics Korea's mortality data is a compilation of death reports acquired from Eup, Myeon, Dong administrative welfare centers, and Si/Gu offices in the country (foreign missions for overseas Koreans) based on resident registration sites.

In this study, MDR-TB patients and DS-TB patients were selected as participants. The description of the codes and criteria for selecting participants are as follows. Participants diagnosed with TB-related ICD-10 codes were selected (A15-A19, U84.30, and U84.31). MDR-TB patients were diagnosed with U84.30 (MDR-TB), U84.31 (XDR-TB), and rifampin-only resistant TB, while other participants were diagnosed with DS-TB. The U84.30 code is assigned to patients with TB bacteria resistant to two or more anti-TB drugs, including isoniazid and rifampin. The U84.31 code is for TB patients resistant to isoniazid and rifampin, one or more quinolone drugs, and one or more of the three injections, capreomycin, kanamycin, and amikacin.

### Incidence-Based Disability-Adjusted Life Years Approach Methods

The disability-adjusted life years (DALY) approach was used as a methodology in this research, and the incidence-based DALY approach was measured by totaling the values corresponding to years lived with disability (YLD) and years of life lost (YLL). One DALY indicates that 1 year of full health was lost to disease, disability, and early death; as DALYs increase, the gap with the ideal health level widens, which suggests an increase in disease burden. The incidence-based DALY approach was measured by totaling the values corresponding to YLD and YLL, and detailed calculation methods are shown in Yoon and Yoon (20). To calculate YLD, the DISMOD II program should be used. The program helps determine the age of incidence and duration of TB. Upon inputting the values for incidence, fatality, and mortality rates into the program, the age and duration of TB incidence are automatically calculated. The DISMOD II program was developed by WHO and has been used since the Global Burden of Disease (GBD) 2000, and the DISMOD II program makes it easy to change the number of age groups for variables as needed. This is very useful when the epidemiological variables are listed in a different age group from the age group trying to represent the YLD results (21). The DISMOD II program is used to measure the incidence-based DALY approach, and detailed methods of calculation are described elsewhere (22). To measure YLL, mortality data for TB (A15–A19) and life expectancy by age, gender, and year should be used using Statistics Korea's statistical yearbook for the cause of death. This will help measure the burden of TB disease in Korea (23, 24).

The incidence rate was primarily measured using NHI claims data, which generated YLD values. Mortality and fatality rates were measured using Statistics Korea's statistical yearbook for the cause of death. To measure YLL, the mortality rate and expected life expectancy for TB patients by age, gender, and year were measured using the Korean life table and the statistical yearbook (25). Cause-specific disability weights (DW) were 0.318 and 0.434 for DS-TB and MDR-TB, respectively. To calculate YLD, YLL, and DALYs per 100,000 people, the mid-year population data by age, gender, and year from Statistics Korea were used (26).

### Economic Burden

The economic burden was measured as the sum of direct and indirect costs for TB patients. For this calculation, NHI claims data and Korean health panel data were used. Direct costs were classified as medical and non-medical costs. In medical costs, insured medical costs include the total inpatient/outpatient medical and drug expenses, while uninsured medical costs include costs of service not covered by insurance. Furthermore, non-medical costs include transportation and caregiver costs (Table 1).

**Table 1:** Economic disease burden variables.

**Variables**			**Detailed Variables**
Direct cost	Medical cost	Insured medical cost	Inpatient, outpatient, drug cost
		Uninsured medical cost	Proportion of non-covered services expenditure
	Non-medical cost	Transportation cost	Number of outpatient visits Average transportation cost by disease category
		Caregiver cost	Hospitalization Average day caregiver cost
Indirect cost	Productivity loss due to morbidity		Number of outpatient visits Hospitalization Average daily income
	Productivity loss due to premature mortality		Number of deaths Average annual income

Indirect cost refers to productivity loss due to morbidity and premature mortality. Productivity loss due to morbidity includes the loss of time in outpatient visits and hospitalization, as well as loss of the average daily income. Subsequently, productivity loss due to premature mortality involves the number of deaths, life expectancy, and average annual income. Indirect cost also includes the loss of productivity due to use of hospital medical and loss of future income as a result of early death.

Indirect cost loss appears to be a social loss, but the Health Insurance Review & Assessment Service (HIRA) does not generally implement policies that take into account indirect costs, and therefore the impact of TB is underestimated. With similar policies, in the case of rare diseases, medical institutions that can be diagnosed as well as treated continuously are concentrated in the metropolitan area, thus, patients living in rural areas were burdened with travel costs. The government is helping people with rare diseases to manage them by consulting, diagnosing and supporting patients with such diseases in the community. It is creating an education and request system through the establishment of a network of local medical services (27).

### Ethics Statement

This study was approved by the Institutional Review Board of National Evidence-based Healthcare Collaborating Agency (IRB No. NECAIRB19-008, NECAIRB20-003, NECAIRB21-013). Informed consent was not required because public data from the NHIS database of de-identity were used.

## Results

### Years Lived With Disability, Years of Life Lost, and Disability-Adjusted Life Years Due to DS-TB and MDR-TB

According to the data on DS-TB patients in the year 2017, 59.5% of this patient population was male, while 40.1% was female, and the average age was 59.6 years. In terms of income quintile, medical aid was 9.9%, and regional/workplace subscribers accounted for the majority 90.1%. In the MDR-TB group, 65.1% were men and 34.9% were women, with an average age of 53.5 years. In the income quintile, medical aid was 12.1%, and regional/workplace subscribers accounted for 87.9% (Table 2).

**Table 2:** Characteristics of drug-susceptible tuberculosis and multidrug-resistant tuberculosis patient, 2017.

**Variables**		**Drug-susceptible tuberculosis**		**Multidrug-resistant tuberculosis**		***p*** **-value**
		***N***	**%**	***N***	**%**	
Gender	Male	16,607	59.9	323	65.1	0.0197
	Female	11,096	40.1	173	34.9	
Age		59.59 ± 19.60		53.51 ± 17.66		<0.0001
Income quintile	Medical AID	2,735	9.9	60	12.1	0.0005
	1	4,760	17.2	103	20.8	
	2	5,147	18.6	106	21.4	
	3	5,891	21.3	110	22.2	
	4	7,113	25.7	95	19.2	
	5	2,057	7.4	22	4.4	
Health insurance type	Medical AID	2,735	9.9	60	12.1	0.1004
	Region/Workplace	24,968	90.1	436	87.9	
	Total	27,703	100.0	496	100.0	

**Figure 1:**
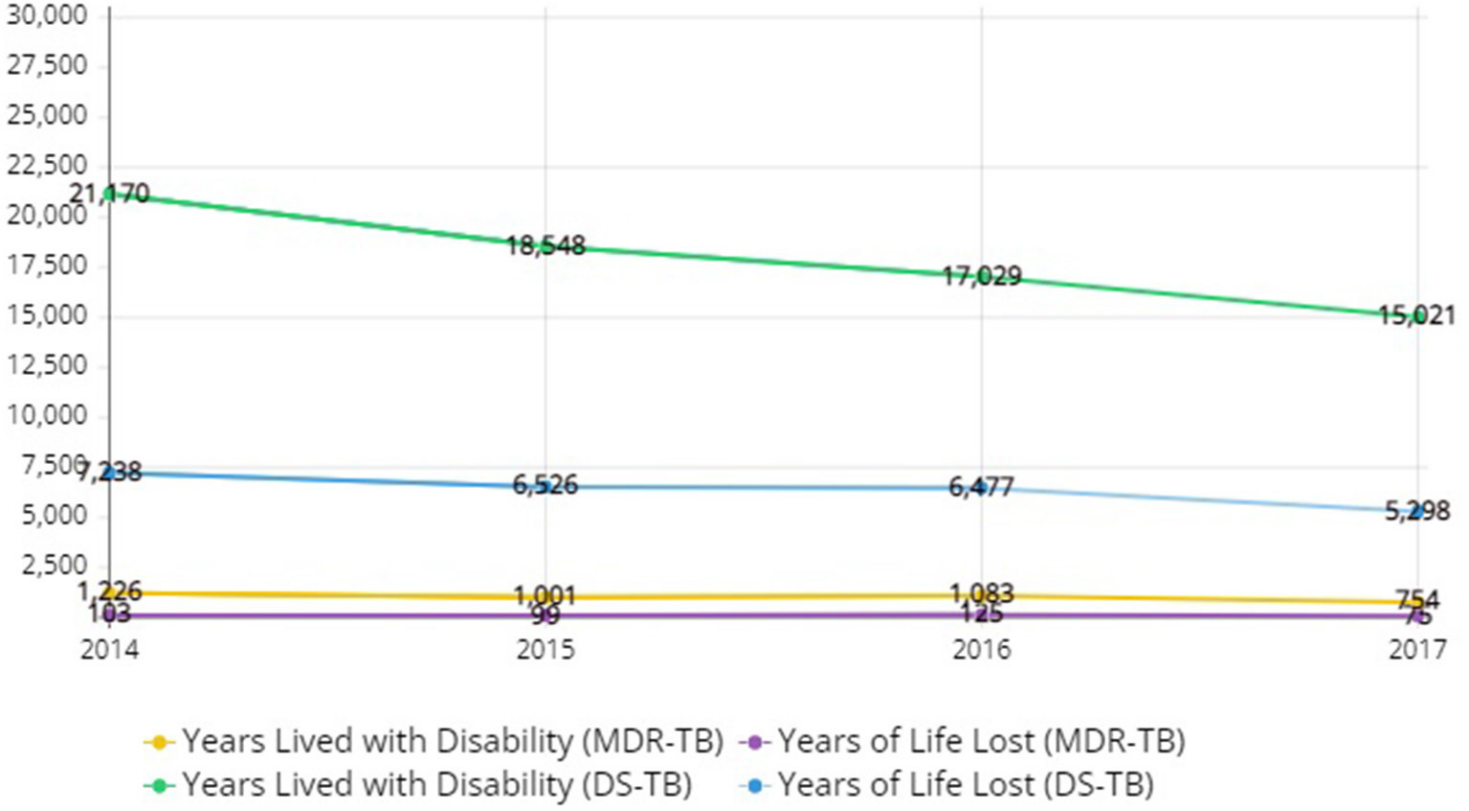
Disability-adjusted life years, years of life lost, years lived with disability due to drug-susceptible tuberculosis and multidrug-resistant tuberculosis in Korea, 2014–2017.

Figure 1 shows the YLL, YLD, and DALY of DS-TB and MDR-TB from 2014 to 2017. DALY is the sum of YLL and YLD. With regard to DALY of DS-TB, there is a decrease from 28,408 DALYs in 2014 to 20,319 DALYs in 2017. MDR-TB also decreased from 1,329 DALYs in 2014 to 829 DALYs in 2017. A consistent decreasing trend for YLL and YLD can be noticed in the figure.

In DALYs per 100,000 people with DS-TB, the values were higher, as the data of an older population was considered. In terms of total DALYs for men and women, it was found to have decreased by 29% from 56 in 2014 to 40 in 2017. Overall, men with DS-TB had higher DALYs than their female counterparts. For most men with MDR-TB in all age groups, DALYs corresponded to 1 in 2017, a slight decrease from 2014. Contrary to the case of men, in women, DALYs appeared to be inconsistent across all age groups. The overall DALYs for men and women were found to have decreased by 33% from 3 in 2014 to 2 in 2017. Overall, no significant gender difference was found in DALYs for MDR-TB patients (Table 3).

**Table 3:** Disability-adjusted life years (DALYs) due to drug-susceptible tuberculosis (DS-TB) and multidrug-resistant tuberculosis (MDR-TB) by gender and age, DALYs per 100,000 people, 2014–2017.

**Variables**		**DS-TB DALYs**				**MDR-TB DALYs**				**DS-TB DALYs per 100,000**				**MDR-TB DALYs per 100,000**			
		**2014**	**2015**	**2016**	**2017**	**2014**	**2015**	**2016**	**2017**	**2014**	**2015**	**2016**	**2017**	**2014**	**2015**	**2016**	**2017**
Male	0–9	152	109	57	52	4	–	–	–	6	5	2	2	0	–	–	–
	10–19	1,624	1,491	1,081	882	47	47	35	23	51	49	37	32	1	2	1	1
	20–29	2,348	1,938	1,598	1,381	68	78	81	40	68	55	45	39	2	2	2	1
	30–39	1,858	1,716	1,467	1,225	78	42	75	39	46	44	38	32	2	1	2	1
	40–49	2,909	2,314	2,300	1,967	71	33	44	47	65	52	52	45	2	1	1	1
	50–59	3,244	3,184	2,855	2,601	54	65	66	54	81	78	69	62	1	2	2	1
	60–69	1,851	1,809	1,830	1,600	30	58	37	17	84	77	73	60	1	2	1	1
	70–79	2,006	1,702	1,722	1,533	30	12	16	10	154	127	126	107	2	1	1	1
	≥80	848	846	1,052	911	4	3	2	6	236	212	239	189	1	1	0	1
Female	0–9	42	20	22	22	–	–	–	–	2	1	1	1	–	–	–	–
	10–19	881	735	586	479	49	16	41	16	30	26	22	19	2	1	2	1
	20–29	1,888	1,572	1,528	1,150	211	219	244	97	60	50	48	36	7	7	8	3
	30–39	1,626	1,409	1,149	1,001	332	287	276	177	42	37	31	28	9	8	7	5
	40–49	1,432	1,122	1,076	930	236	104	123	151	33	26	25	22	5	2	3	4
	50–59	1,288	1,129	950	912	54	58	96	74	32	28	23	22	1	1	2	2
	60–69	981	771	800	752	29	37	34	42	42	31	30	27	1	1	1	2
	70–79	2,129	1,855	1,859	1,501	26	35	26	21	120	103	103	81	1	2	1	1
	≥80	1,302	1,354	1,573	1,420	8	5	13	14	154	148	160	135	1	1	1	1
Male Total		16,839	15,108	13,963	12,152	385	338	356	237	66	59	55	48	2	1	1	1
Female Total		11,569	9,965	9,542	8,167	944	762	853	593	46	39	37	32	4	3	3	2
Total		28,408	25,074	23,505	20,319	1,329	1,099	1,208	829	56	49	46	40	3	2	2	2

### Economic Burden Due to DS-TB and MDR-TB

**Figure 2:**
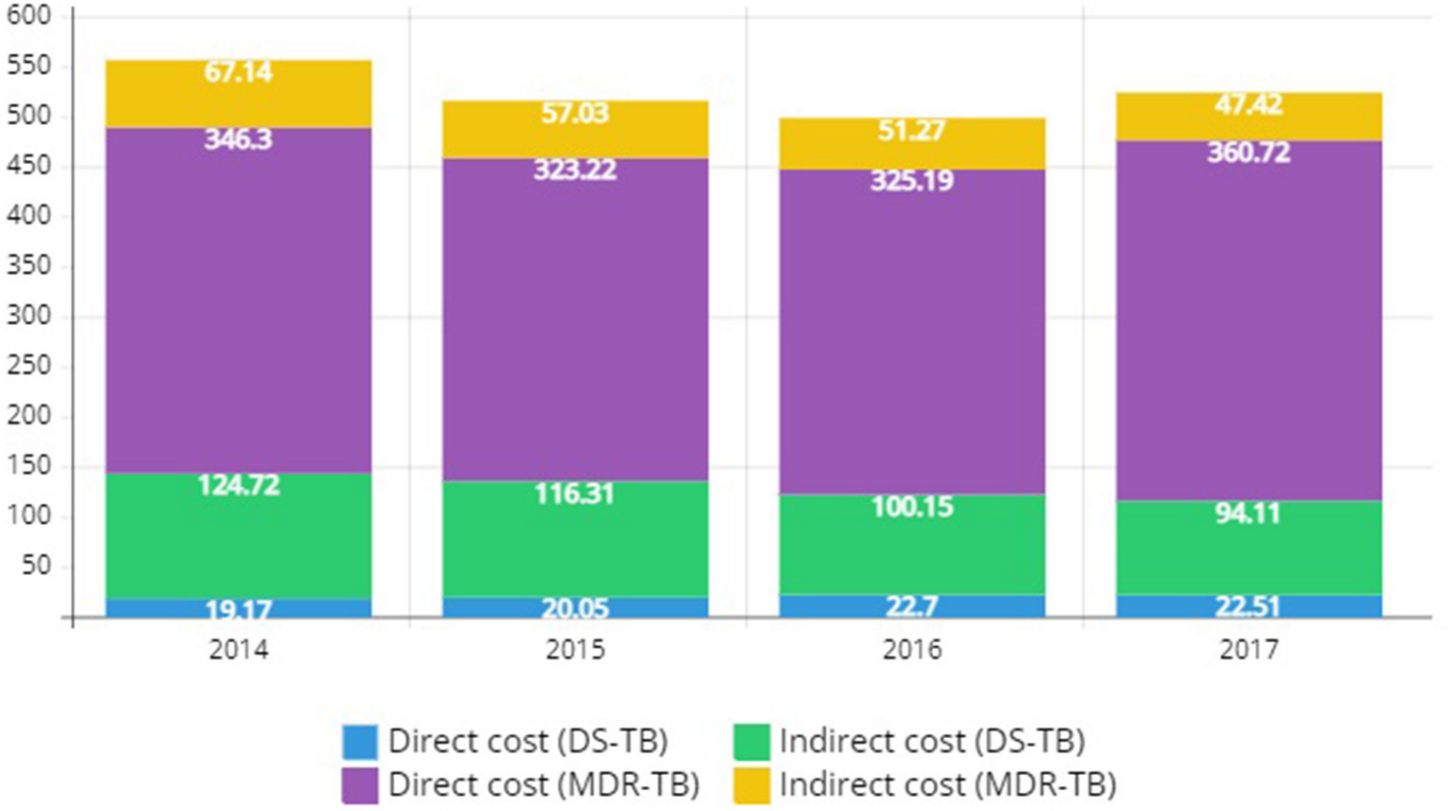
Economic disease burden of drug-susceptible tuberculosis and multidrug-resistant tuberculosis in Korea, 2014–2017 (Discount rate: 3%, Unit: $1 million).

Figure 2 shows the direct cost, indirect cost, and total cost of DS-TB and MDR-TB from 2014 to 2017. The total cost of DS-TB decreased from $143.89 million in 2014 to $116.62 million in 2017. The total cost of MDR-TB decreased slightly from $413.44 million in 2014 to $408.14 million in 2017. Nevertheless, it was still burdensome.

The total cost of DS-TB in 2017 was the highest among men in their 40s and 50s. This parameter was relatively similar among women for all age groups. The total cost for both men and women experienced a 19% decrease from $143.89 million in 2014 to $116.62 million in 2017. MDR-TB was high for men in their 40s and older for women in their 70s and older. The total cost for men and women showed a slight decrease from $413.44 million in 2014 to $408.14 million in 2017. The decrease in death cost as part of indirect costs is believed to be the reason for this decline (Table 4).

**Table 4:** Total cost of drug-susceptible tuberculosis (DS-TB) and multidrug-resistant tuberculosis (MDR-TB) by gender and age, 2014–2017 (Discount rate: 3%, Unit: $1 million).

**Variables**		**DS-TB total cost**				**MDR-TB total cost**			
		**2014**	**2015**	**2016**	**2017**	**2014**	**2015**	**2016**	**2017**
Male	0–9	0.01	–	0.07	–	0.19	0.09	0.03	0.05
	10–19	0.27	0.10	0.08	0.25	2.33	2.09	1.59	1.59
	20–29	4.28	2.61	1.59	3.73	10.66	8.61	8.94	8.34
	30–39	9.69	10.19	6.27	5.23	18.81	14.49	13.76	12.92
	40–49	42.70	33.24	32.01	35.84	47.16	35.37	32.12	35.67
	50–59	50.43	53.10	44.27	41.60	75.50	71.44	65.07	61.70
	60–69	15.77	16.02	17.19	13.97	47.87	47.13	46.62	54.86
	70–79	1.35	1.32	1.10	1.83	45.29	40.48	42.36	50.12
	≥80	0.47	0.29	0.54	0.88	26.69	28.59	33.16	40.56
Female	0–9	–	–	–	–	0.16	0.03	0.03	0.08
	10–19	0.07	0.02	0.06	0.03	1.61	1.47	0.98	1.15
	20–29	2.01	2.46	4.10	0.88	9.06	6.73	6.26	7.06
	30–39	4.27	3.92	1.52	1.18	10.79	8.63	7.75	7.52
	40–49	3.81	4.16	4.21	2.58	11.22	10.23	9.82	8.51
	50–59	4.38	5.34	4.55	3.60	14.75	13.34	12.52	12.13
	60–69	2.81	1.78	2.56	2.23	15.29	16.03	14.53	17.13
	70–79	1.01	0.98	1.42	1.41	36.52	34.54	33.98	34.41
	≥80	0.56	0.83	1.30	1.39	39.54	40.96	46.94	54.33
Male Total		124.97	116.87	103.12	103.32	274.51	248.29	243.65	265.82
Female Total		18.91	19.49	19.73	13.29	138.94	131.96	132.81	142.32
Total		143.89	136.36	122.85	116.62	413.44	380.25	376.46	408.14

In total cost by category, the direct cost of DS-TB in 2017 was $22.51 million, and the indirect cost was $94.11 million. This gave a total cost of $116.62 million. Medical costs account for a high percentage of direct costs, and productivity loss due to premature mortality account for 96% of indirect costs. From 2014 to 2017, the total cost showed a decreasing trend, which was estimated to be due to a decrease in death cost. The direct cost of MDR-TB in 2017 was $360.72 million and the indirect cost was $47.42 million, which gives the total cost of $408.14 million. Hospitalization cost constituted 60% of the direct cost, outpatient cost 31%, and care cost 8%. Productivity loss due to morbidity accounted for 96% of the indirect costs, which contradicts the finding for DS-TB. Compared to 2014, the total cost decreased slightly in 2017, which is estimated to be a result of a decrease in disease costs as part of indirect costs (Table 5).

**Table 5:** Total cost of drug-susceptible tuberculosis (DS-TB) and multidrug-resistant tuberculosis (MDR-TB) by category, 2014–2017 (Discount rate: 3%, Unit: $1 million).

**Variables**			**DS-TB total cost**				**MDR-TB total cost**			
			**2014**	**2015**	**2016**	**2017**	**2014**	**2015**	**2016**	**2017**
Direct cost	Total		19.17	20.05	22.70	22.51	346.30	323.22	325.19	360.72
	Medical cost	Insured medical cost	10.50	10.96	12.31	11.30	200.36	186.92	186.91	214.99
		Uninsured medical cost	6.70	7.14	8.05	9.58	114.96	103.83	105.16	113.23
	Non-	Transportation cost	0.14	0.14	0.15	0.12	1.92	1.94	1.96	1.96
	medical cost	Caregiver cost	1.83	1.82	2.20	1.51	29.06	30.53	31.16	30.55
Indirect cost	Total		124.72	116.31	100.15	94.11	67.14	57.03	51.27	47.42
	Productivity loss due to morbidity		6.99	6.02	6.18	4.21	63.76	53.54	46.38	45.62
	Productivity loss due to premature mortality		117.72	110.30	93.97	89.90	3.39	3.49	4.89	1.80
Total cost			143.89	136.36	122.85	116.62	413.44	380.25	376.46	408.14

## Discussion

This study measured the change in the status of TB burden for DS-TB and MDR-TB in Korea from 2014 to 2017 and evaluated the disease burden for each age group and gender. DS-TB-related DALYs per 100,000 people were found to have decreased over the study period (59, 49, 46, and 40, respectively). Subsequently, DALYs per 100,000 people for MDR-TB slightly decreased and then sustained during the observed period (3, 2, 2, and 2, respectively). The economic burden of DS-TB consistently reduced from 2014 to 2017, while that of MDR-TB fluctuated each year with no specific trend.

DALYs per 100,000 people showed a decreasing trend for DS-TB and MDR-TB. In 2017, the DALYs per 100,000 people were 40 for DS-TB and 2 for MDR-TB. By gender, DALYs per 100,000 people was higher in men than those in women with DS-TB (men 48, women 32), but for MDR-TB, they were no significant difference between men and women (men 1, women 2). Previous study also showed a trend of decreasing DALYs per 100,000 people of general TB from 2014 to 2017, with 63 DALYs per 100,000 people for men and 36 DALYs per 100,000 people for women in 2017 (25). By age, DALYs per 100,000 people increased for DS-TB with an increase in age. For MDR-TB, it was found to be higher in middle-aged patients. In a 2015 study on the disease burden on Korean patients, TB DALYs per 100,000 people was 121 for men and 76 for women, and DALYs per 100,000 people for male patients aged 80 or older was approximately 92% higher than that for female patients (28). In a 2017 TB study, DALYs per 100,000 people aged 80 or older was 279 males and 166 females, about 68% higher in males than females (25). In this study, men over 80 years of age showed 40% higher than women in DS-TB DALYs per 100,000 people (men 189, women 135). However, in MDR-TB DALYs per 100,000 people, there was no difference between men and women over 80 years of age (men 1, women 1).

In a 2015 study of economic burden, the total cost of TB was found to be $616.80 million, with a direct cost of $199.40 million and an indirect cost of $417.40 million (28). In this study, the total cost of DS-TB had a decreasing trend, whereas the total cost of MDR-TB was different for each year, with no specific trend. The difference between the total costs for DS-TB and MDR-TB tended to increase every year. In 2014, the total cost of MDR-TB was 2.9 times higher than that of DS-TB, whereas, in 2017, it was 3.5 times higher. This is because the total cost of MDR-TB was higher for patients aged more than 80.

According to the GBD study, the total DALYs for infectious diseases in 2017 was 6.5% of that of TB for all age groups, which is higher for patients aged 65 or above at 17% (29). Statistics Korea predicts that the older population, aged 65 years or older, in the country will increase by 46% in the next 50 years (24). With the rapid growth of the aging population, a high burden of TB on the older is expected in the future. Thus, it is necessary to prioritize TB management among this older population in Korea.

MDR-TB is a central issue in TB management due to its low treatment success rate, high mortality rate, and high disease burden. To increase the treatment success rate of MDR-TB, early diagnosis, appropriate treatment, and effective patient management must be accomplished by the national TB management system; there must be an efficient workforce capable of integrating and managing these aspects with the larger system (30). MDR-TB is declining in Korea. To increase the treatment success rate of MDR-TB, treatment support from the NHIS was initiated in 2014 by including TB as a rare and intractable disease to ensure that patients are eligible to receive reimbursement for treatment expenditure (31), and health insurance benefits. As a result, patients have been exempted from economic burden since July 2016.

Meanwhile, the influx of foreign TB, especially MDR-TB patients, is increasing due to an increase in the inflow of people in Korea from high-risk countries for TB on the account of employment, education, and migration (32). Furthermore, the settlement of North Korean defectors has also increased (33). They need appropriate management interventions since they might become an undiscovered source of infection as a result of frequent movement within groups and limited access to medical due to their illegal stay in the country (34, 35).

One study limitation was that fewer MDR-TB deaths were selected due to limitations in data sources. This is because MDR-TB deaths in the current year were analyzed in the study. Consequently, the YLL of MDR-TB was lower than that of DS-TB. However, these limited data alone confirmed that the DALYs of MDR-TB was low. In future studies, it will be necessary to select the data source to include a wide range of MDR-TB patients as subjects.

Despite these limitations, the significance of this study is that it can be used as the basis for understanding the disease burden of TB by comparing DALYs and economic burden according to the presence or absence of drug resistance with the use of integrated data. Due to limitations in data sources and difficulties in selecting MDR-TB subjects, the current research on health outcomes using standardized methodologies is insufficient. However, this paper can be used as a reference study.

Through this study, DALYs and economic burden on patients were compared for the study period of 2014–2017 according to TB resistance (DS-TB and MDR-TB). It was found that the total cost for DS-TB decreased and MDR-TB caused a huge social burden. Although the number of MDR-TB patients was fewer than that of DS-TB, the cost burden was extremely high for MDR-TB. In society, the aging population is rapidly increasing. Therefore, the incidence and spread of TB in all areas of society can be suppressed through intensive management of MDR-TB in the older population.

To reduce the threat of early community transmission and resistance development for TB, public-private mixed collaboration for TB management was launched in 2009 (10). The main elements of this collaboration are strict monitoring of patients, investigation of close contacts, and financial support for patients. In the same context, The Korea Centers for Disease Control & Prevention has implemented a review process for the approval of new TB drugs used to treat patients with multidrug-resistant tuberculosis (MDR-TB) since September 2016 to prompt initiation of MDR TB treatment to reduce early community transmission (36). In addition, Korean government authorities are performing the active TB screening of elderly people even though concerns related to cost-effectiveness and medical issues like morbidity remain (37).

In particular, national-level MDR-TB patient management and support are required to reduce the number of TB deaths. While implementing a comprehensive TB management plan in Korea, the allocated budget should be increased based on the priorities. We hope that the national TB management plan will soon be efficiently implemented given the fact that the country's infectious disease management system is currently prioritizing the management of the COVID-19 over other diseases.

## Data Availability Statement

Publicly available datasets were analyzed in this study. This data can be found here: https://nhiss.nhis.or.kr/bd/ay/bdaya001iv.do. This study used the National Health Information Database (NHIS-2019-1-662) of the National Health Insurance Service (NHIS).

## Ethics Statement

The studies involving human participants were reviewed and approved by National Evidence-Based Healthcare Collaborating Agency. Written informed consent from the participants' legal guardian/next of kin was not required to participate in this study in accordance with the national legislation and the institutional requirements.

## Author Contributions

SL, MJK, and I-HO: conceptualization, data curation, and writing—original draft. SL and I-HO: writing—review and editing. SHL, H-YK, H-SK, and I-HO: supervison. All authors contributed and approved the article.

## Funding

This study was financially supported by the National Evidence-based Healthcare Collaborating Agency, funded by the Ministry of Health and Welfare (Grant Nos. NC19-002, NC20-003, and NC21-001), and by an Intramural Research grant from the Korean National Tuberculosis Association.

## Conflict of Interest

The authors declare that the research was conducted in the absence of any commercial or financial relationships that could be construed as a potential conflict of interest.

## Publisher's Note

All claims expressed in this article are solely those of the authors and do not necessarily represent those of their affiliated organizations, or those of the publisher, the editors and the reviewers. Any product that may be evaluated in this article, or claim that may be made by its manufacturer, is not guaranteed or endorsed by the publisher.
